# Case Report: Pediatric Renal Sarcoidosis and Prognostic Factors in Reviewed Cases

**DOI:** 10.3389/fped.2021.724728

**Published:** 2021-09-13

**Authors:** Richard Klaus, Annette Friederike Jansson, Matthias Griese, Tomas Seeman, Kerstin Amann, Bärbel Lange-Sperandio

**Affiliations:** ^1^Division of Pediatric Nephrology, Department of Pediatrics, Dr. v. Hauner Children's Hospital Ludwig-Maximilians University, Munich, Germany; ^2^Department of Pediatrics, Dr. v. Hauner Children's Hospital Ludwig-Maximilians University, Munich, Germany; ^3^German Center for Lung Research (DZL), Munich, Germany; ^4^Institute for Pathology, University Hospital Erlangen, Erlangen, Germany

**Keywords:** sarcoidosis, tubulointerstitial nephritis, acute kidney injury, case report, prognostic factor analysis

## Abstract

**Background:** Pediatric sarcoidosis is a complex inflammatory disorder with multisystemic manifestations. Kidney involvement in children is rare, and prognostic factors are unknown.

**Case Report and Methods:** We report the case of a 16-year-old girl with multiorgan sarcoidosis and renal involvement. The patient presented with tubulointerstitial nephritis, acute kidney injury (AKI), chest CT disseminated noduli, granulomatous iridocyclitis, giant-cell sialadenitis, and arthralgia. The kidney biopsy revealed non-granulomatous interstitial nephritis. Treatment consisted of initial high-dose methylprednisolone pulse followed by oral prednisolone and methotrexate. Full remission was achieved. In addition, we performed a literature review using PubMed and analyzed data on pediatric renal sarcoidosis cases.

**Results:** We identified 36 cases of pediatric sarcoidosis with renal involvement on presentation and data on the end-of-follow-up glomerular filtration rate (GFR). The data from the literature review showed that renal involvement was slightly more prevalent in males (60%). AKI was present in most of the described patients (84%). Oral prednisolone was used in 35 of 36 cases; in more severe cases, other immunosuppressants were used. We newly identified renal concentration impairment and granulomatous interstitial nephritis as factors with a clear trend toward GFR loss at the end of follow-up, emphasizing the importance of kidney biopsy in symptomatic patients. In contrast, higher GFR at presentation and hypercalcemia were rather favorable factors. According to the identified predictive factors, our patient has a good prognosis and is in remission.

**Conclusion:** The factors indicating a trend toward an unfavorable renal outcome in pediatric sarcoidosis are renal concentration impairment and granulomatous interstitial nephritis at presentation, while a higher GFR is beneficial.

## Introduction

Sarcoidosis is a systemic inflammatory disease characterized by the formation of non-necrotizing epithelioid cell granulomas with multiorgan affection. The reported incidence is 0.29–0.8 per 100,000 children (1–3). While adult sarcoidosis is often diagnosed in asymptomatic patients by routine chest x-ray, 66–98% of children present with acute symptoms such as fever, fatigue, and multiorgan involvement (1–3). Common manifestations include uveitis/iritis, hilar and peripheral lymphadenopathy, hepatosplenomegaly, arthritis, parenchymal lung disease, and skin rash. To date, the pathophysiology of this multifactorial disorder remains elusive. A combination of genetic factors (e.g., CARD15/NOD2-mutation) and environmental exposure with antigens such as infectious agents—mainly mycobacteria (4), silica, aluminum, or inorganic dusts (5)—is suspected to trigger a CD4+ T-cell response with increased cytokine activities resulting in granuloma formation. Prolonged inflammation may lead to fibrosis and irreversible organ dysfunction (6). Autonomous expression of 1-alpha-hydroxylase and therefore overproduction of 1,25-vitamin D in activated macrophages may result in hypercalcemia and secondary nephrocalcinosis (7). Histological evidence of non-necrotizing epithelioid granulomas with giant cells in affected organs and exclusion of other diagnoses (especially granuloma-causing infections, immunodeficiencies, and oncological entities) establishes the diagnosis. Therapeutic regimens mostly include steroids and add other immunosuppressants either as steroid-sparing agents or in refractory cases (8).

There are a few collections of cases of pediatric renal sarcoidosis, and so far, no attempt to analyze prognostic factors has been made. To our knowledge, we here present the largest number of collected previously published cases on renal sarcoidosis in children.

## Case Report

**Figure 1:**
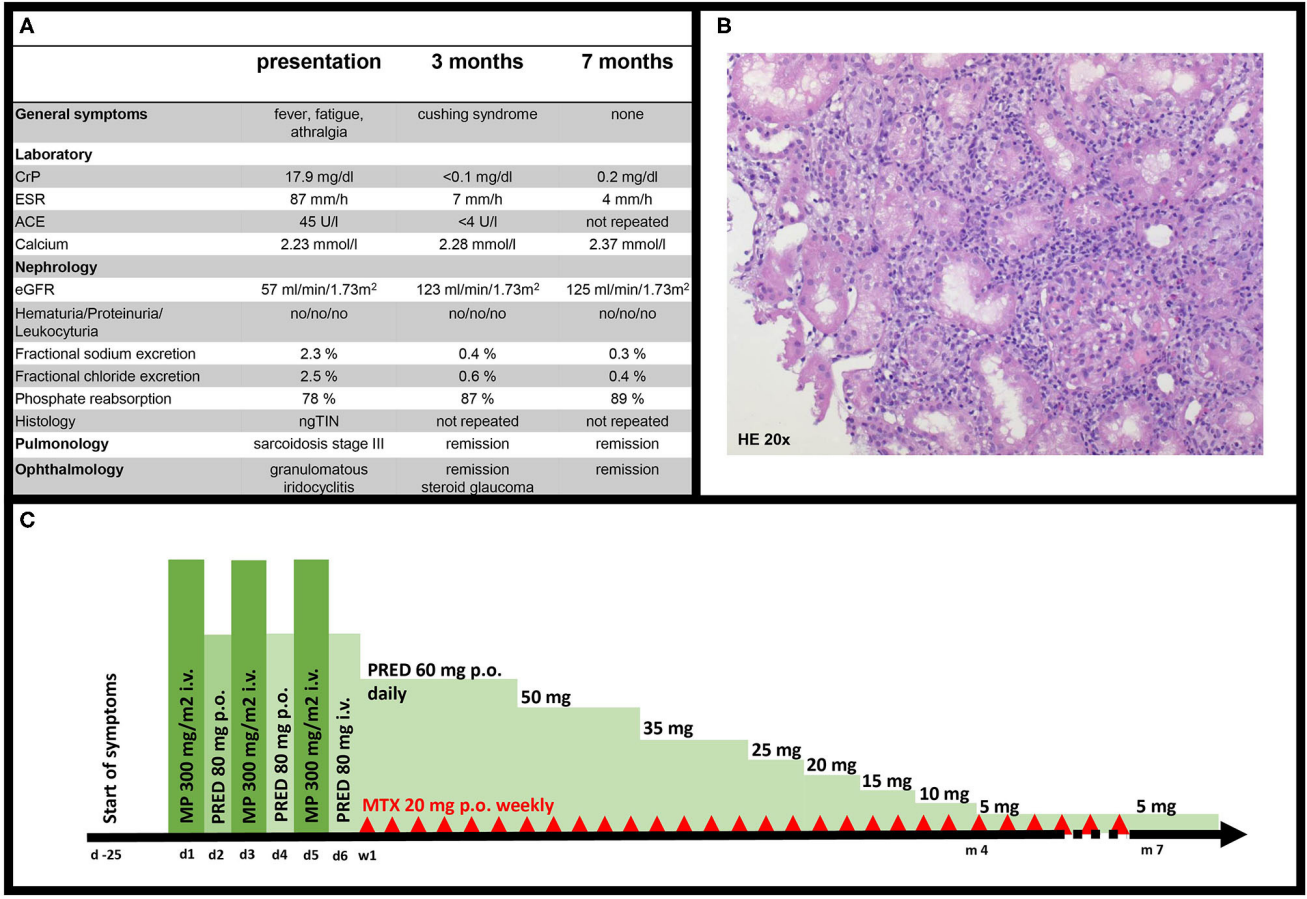
**(A)** Clinical characteristics of our patient at presentation, 3 and 7 months after therapy initiation. **(B)** ngTIN in our patient, 20x magnification, hematoxylin and eosin (HE) stain. **(C)** Course of treatment in our patient. d, day; w, week; m, months; MP, methylprednisolone; PRED, prednisolone; MTX, methotrexate.

A 16-year-old girl (52 kg, 158 cm) with no relevant medical history was admitted to our hospital with a 9-day history of bilateral cervical mass, fever resistant to antibiotics, fatigue, and night sweat with suspicion of lymphoma. Examination showed a stable cardiorespiratory condition (blood pressure: 107/70 mmHg) with bilateral, indolent, firm and immobile submandibular mass, and arthralgia in both knees. Vision was mildly blurred; macroscopically, no eye abnormalities were present. Basic laboratory studies including blood count and blood gas analysis were normal besides elevated inflammatory parameters [C-reactive protein (CrP), 17.9 mg/dl; erythrocyte sedimentation rate (ESR), 87 mm/h] (Figure 1A). Chest x-ray showed disseminated nodules and interstitial consolidations. Lymphoma was suspected, and a PET-CT showed increased metabolic activity in the suspected lymph nodes, kidneys, lungs, bronchi, spleen, and bone marrow. Surprisingly, the cervical mass biopsy showed no signs of malignancy, but sialadenitis with epithelioid and giant cells. Acute kidney injury (AKI) developed (KDIGO stage 1), and glomerular filtration rate (GFR) was 57 ml/min/1.73 m^2^ according to Schwartz formula (9). Maximum serum creatinine was 1.5 mg/dl, and cystatin C was 1.48 mg/l. Urea and electrolytes were in the normal range. There was no proteinuria, no hematuria, and no leukocyturia. Hemoglobin concentration and thrombocyte count were normal, lactate dehydrogenase was not elevated, and complement level was normal. Urinary calcium excretion was within normal limits (1.04 mg/kg/d, *N* <4 mg/kg/d). Tubulointerstitial nephritis (TIN) with tubular salt wasting (fractional sodium excretion: 2.3%, fractional chloride excretion: 2.5%, and phosphate reabsorption: 78%) and impaired concentration capacity (tubular reabsorption of water 97%, calculated by: (1 – (creatinine_serum_/creatinine_urine_) × 100 *N* >98.5%) were diagnosed. Although the most frequent causes for TIN are drugs and infections, a systemic disease was suspected as the cause for TIN. Ophthalmologic evaluation was performed because of mildly blurred vision and because of suspicion of systemic disease. Presence of granulomatous iridocyclitis suggested sarcoidosis. Kidney biopsy, chest CT, and bronchoscopy with bronchoalveolar lavage (BAL) were performed. The kidney biopsy showed interstitial nephritis with predominantly CD3-positive T cells, but also granulocyte infiltration and acute tubular epithelial damage (Figure 1B). Epithelioid granulomas or necrosis were not found. Glomeruli (including immunohistochemistry for IgA, IgM, C1q, and C3c) and vessels were unaffected; electron microscopy was unremarkable. Bronchoscopy showed granular inflammation of the mucosa with epithelioid cellular granulomas with giant cells. BAL showed predominantly T-cellular inflammation (87%) with a normal CD4/CD8 ratio (3.2, *N*: 1.0–3.6). Pulmonary function was normal, and chest CT showed disseminated nodules and ground-glass opacity. Pulmonary findings were compatible with sarcoidosis stage III. Treatment was initiated with 300 mg/m^2^ methylprednisolone intravenously on days 1, 3, and 5 with 80 mg prednisolone orally on days 2, 4, and 6. From day 7, oral prednisolone was continued with 60 mg daily. Oral methotrexate (20 mg weekly) was used because of the multiorgan involvement. Prednisolone was slowly tapered, aiming for a 6-month period to achieve the maintenance dose of 5 mg daily (Figure 1C). One month after therapy initiation, kidney function had already normalized (creatinine, 0.8 mg/dl; GFR, 108 ml/min/1.73 m^2^; and fractional sodium excretion, 0.9%). After remission, follow-up was continued every 2 months with physical examination, urine analysis, and laboratory studies of inflammatory markers. Because of stable remission and adverse effects of steroids (transiently increased intraocular pressure, cushing syndrome, hair loss, and tremor), tapering was accelerated to reach maintenance dose of 5 mg daily, and methotrexate was terminated. Currently, 7 months after the diagnosis, she is in complete remission with no systemic signs of inflammation and a normal GFR.

### Patients and Methods

Here, we use a single case for illustration of renal involvement in sarcoidosis as presenting organ and compared it with previously published pediatric cases. We performed a literature review using PubMed with the terms “renal,” “kidney,” “pediatric,” and “sarcoidosis” in various combinations to collect clinical data from published cases of pediatric sarcoidosis in the years 1999–2021. Thirty-six were identified. Pediatric patients with renal sarcoidosis and a specified or calculated GFR at end of follow-up were included. Renal sarcoidosis was defined as histologically proven sarcoidosis in combination with either a renal histology result or with AKI. All cases were already published in smaller series (1, 10–12). Three cases were reported from the pediatric sarcoidosis register in Louisiana (1). Two were excluded because no end-of-follow-up GFR was available. A series from Paris by Coutant et al. identified 11 cases of renal involvement in children with sarcoidosis and additionally summed up 19 single-case reports. These 19 cases were also included (12). Where possible, additional data were compiled from the original reports; otherwise, data from the collection of Coutant were taken (12). Two cases from the collection of Coutant were excluded because neither biopsy data nor end-of-follow-up GFR was available; two cases were excluded because our criteria for renal sarcoidosis were not met. Hobbs et al. reported one case and reviewed two cases (11). Wang et al. reported one case themselves and reviewed another eight single cases, two overlapping with Hobbs. One was excluded because of no GFR at end of follow-up or serum creatinine was provided (10). Collected data for each patient included age, sex, GFR at presentation, presence of AKI, hematuria, leukocyturia, proteinuria, concentration impairment/polyuria, hypercalcemia, biopsy result, duration of follow-up, and GFR at end of follow-up (Supplementary Table 1). Biopsy result was categorized into three possible categories: “granulomatous interstitial nephritis (GIN),” “non-granulomatous tubulointerstitial nephritis (ngTIN),” and “other.” If GFR was reported as normal at the end of follow-up but no numeric value was specified, a GFR of 100 ml/min/1.73 m^2^ was assumed for calculations (14 cases). If end-stage renal disease or death was reported, we used a GFR of 0 ml/min/1.73 m^2^ for calculations (five cases). If only serum creatinine was recorded, Schwartz-formula was used (9). If no height was recorded, a height of the 50th percentile was assumed (two cases); if no follow-up height was recorded, growth along the last specified percentile was assumed but adjusted for the increased age at end of follow-up (four cases). Not all case collections or original cases provided definitions for the clinical symptoms such as renal concentration impairment or proteinuria, but sometimes only indicated whether they were present or not. Hence, they were included as they were recorded in the original articles. If polyuria was diagnosed, it was recorded as concentration impairment. Therapy not specified beyond “steroids” was recorded as “oral prednisolone” (three cases).

### Statistical Analysis

To determine the influence on the end-of-follow-up GFR in the reported cases, univariate linear regression was performed using SPSS v27. For AKI, hematuria, proteinuria, leukocyturia, concentration impairment, and hypercalcemia, absence of these symptoms was used as baseline. For the biopsy result, GIN was compared to ngTIN as baseline since all biopsies had an abnormal histology as proof of renal involvement, and from a clinical point of view, ngTIN was considered the less severe histology.

## Results

**Figure 2:**
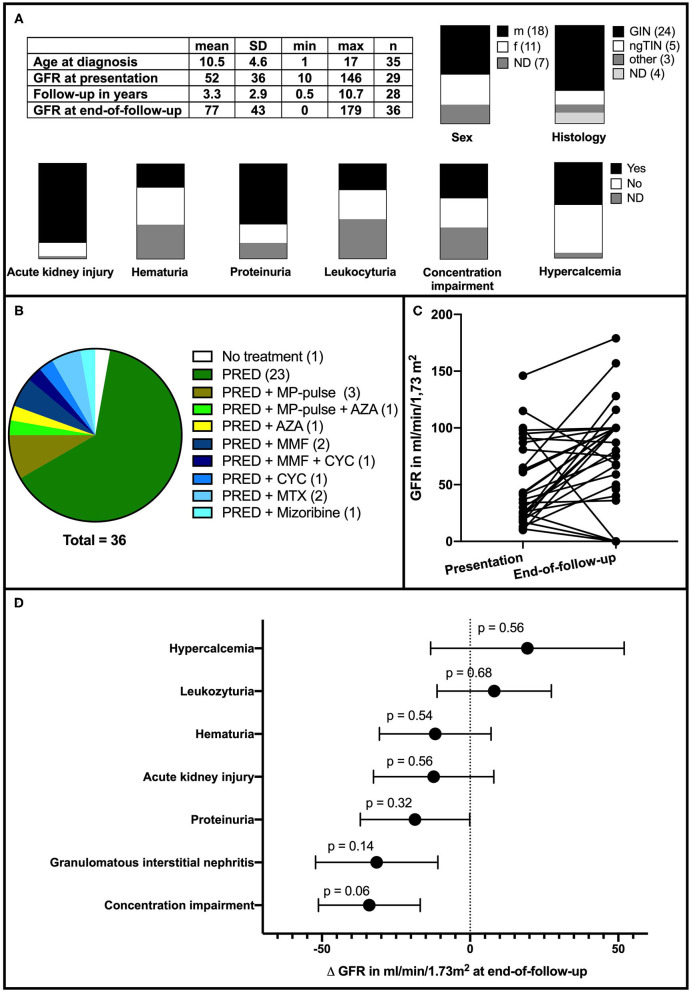
**(A)** Clinical features and symptoms in reported cases (*n* = 36). max, maximum; min, minimum SD, standard deviation; ND, not determined; m, male; f, female. **(B)** Distribution of used medications. PRED, prednisolone; MP, methylprednisolone; AZA, azathioprin; MMF, mycophenolate mofetil; MTX, methotrexate; CTX, cyclophosphamide. **(C)** Course of GFR in the reported cases. **(D)** Influence of initial presentation on GFR at the end of follow-up. GIN opposed to ngTIN and concentration impairment are strongest yet statistically insignificant predictors for a decline in GFR.

A total of 36 cases with pediatric sarcoidosis and renal involvement were included in the statistical analysis (Figure 2A). Data of all included and excluded cases can be found in Supplementary Table 1. Mean age of onset was 10.5 ± 4.6 years. The male-to-female ratio was 18:11 (seven unspecified); hence, 62% of patients with a reported gender were male. The spectrum of renal symptoms was broad and included hematuria (9/23), proteinuria (23/30), hypercalcemia (16/34), leukocyturia (10/21), and impaired renal concentration capacity (13/24). In the majority of cases, AKI was present (30/35) (Figure 2A). GFR at presentation was reported in 29 of 36 cases with a mean of 52 ± 36 ml/min/1.73 m^2^. Renal biopsies mainly revealed GIN (24) or ngTIN (5). Two showed nephrocalcinosis, and one membranous nephropathy; in four cases, no biopsy specimen was obtained. Follow-up period was 3.3 ± 2.9 years (specified in 28/36 cases). The mean GFR at the end of follow-up was 77 ± 43 ml/min/1.73 m^2^. One patient received no treatment because of spontaneous resolution, and all other patients received at least oral prednisolone. Dosages varied, and therapy duration was many months to years. In more severe cases, intravenous methylprednisolone pulse or other immunosuppressants were added (Figure 2B). Under the mentioned therapies, only five patients had a relevant GFR loss, and four progressed to end-stage renal disease (Figure 2C). Concentration impairment (−34 ± 17 ml/min/1.73 m^2^, *p* = 0.06) and GIN in biopsy (−32 ± 21 ml/min/1.73 m^2^, *p* = 0.14) showed trends to negatively predict end-of-follow-up-GFR (Figure 2D). Presence of hypercalcemia showed a trend to positively influence end-of-follow-up GFR (+19 ± 33 ml/min/1.73 m^2^, *p* = 0.56). Another positive predictive feature was the GFR at presentation, which had an unstandardized B-value of 0.43 ± 0.22 ml/min/1,73 m^2^ (*p* = 0.06) in the linear regression. This implies that the end-of-follow-up GFR is improved by 0.43 ml/min/1.73 m^2^ min per 1 ml/min/1.73 m^2^ increased GFR at presentation. Detailed results of the individual univariate linear regressions can be found in Supplementary Table 2.

## Discussion

Childhood sarcoidosis is a granulomatous disease affecting multiple organs. More than 70% of children have 3.8–5.1 organs involved (1, 3). Surprisingly, reported cases of renal involvement in 116 cases of pediatric sarcoidosis are rare and only amount to 3 (1–3). We performed a literature review and found a total of 36 cases of renal involvement to be included into our analysis. We could show that AKI is a common feature (86%) in pediatric sarcoidosis with renal involvement. Other renal symptoms occur, but no single symptom occurs in all children and no specific sign for renal involvement exists. Our findings are consistent with patient characteristics from an adult cohort with 47 patients with renal sarcoidosis. In adults, 62% presented with renal failure with a GFR <30 ml/min/1.73 m^2^, 22% had hematuria (47% in our collection), 29% had leukocyturia (48% in our collection), and 66% had proteinuria (78% in our collection). Hypercalcemia was present in 34% of all cases (47% in our collection) (13). Overall, it appears that children with renal sarcoidosis tend to be more symptomatic than adults. GIN is the predominant histology in adults and children (66% in our collection and 79% in the adult series) (13). Although sarcoidosis in general is evenly distributed among the sexes (8), renal involvement seems to be slightly more prevalent in male patients: 62% in our collection and 60% of patients in the adult collection are male (13). Both the lack of systematic screening and the lack of specific clinical symptoms may account for underreporting of renal sarcoidosis. To improve detection, Bergner et al. suggest a kidney biopsy if AKI, proteinuria, leukocyturia, or hematuria is present. A renal biopsy should be considered in pediatric patients with sarcoidosis and renal symptoms. Presence of non-necrotizing granulomas are necessary to establish the diagnosis. Furthermore, as shown from this case collection, the histology may help to assess the prognosis and to choose the treatment. Nevertheless, in cases with mild renal involvement and an established diagnosis, a renal biopsy can be omitted. As reported in the present case, nephrocalcinosis should be evaluated with a kidney ultrasound and measurement of vitamin D metabolites, serum calcium, and urine calcium concentration (14). Indications for biopsy were AKI and polyuria with salt wasting and impaired concentration ability: hallmarks of TIN. The biopsy revealed the ngTIN.

Prognosis of renal sarcoidosis is difficult to capture and highly variable. So far, no treatment studies exist, and reported case series were relatively small. Full recovery as well as end-stage renal disease are described (12). Predictive parameters would be a key component to adapt the therapy intensity and duration for each individual patient. Hence, we analyzed the predictive value of symptoms at presentation with linear regression. Because of the small number of cases, no clinical feature at presentation could significantly predict outcome, but clear trends exist. The predictors with the strongest trend for decline in GFR are presence of GIN (*p* = 0.14), concentration impairment (*p* = 0.06), and the intensity of GFR impairment at presentation (*p* = 0.06).

Both GIN and concentration impairment represent relevant tubulointerstitial damage; therefore, their positive correlation with a more severe clinical outcome seems reasonable. In contrast, less severely affected patients might not have enough tubulointerstitial damage for clinically apparent concentration impairment. Additionally, granuloma burden in lesser affected patients is probably low, and therefore, granulomas might be missed in the biopsy due to sampling error. This finding is supported by data from an adult case series, where the subgroup of patients with CKD 5 at end of follow-up was exclusively diagnosed with GIN, while that with CKD 1–2 predominantly had ngTIN (15). In contrast, the higher the GFR was at presentation and therefore the lesser the renal damage, the better was the renal outcome in pediatric renal sarcoidosis.

Interestingly hypercalcemia also showed a trend toward a better prognosis. Hypercalcemia in sarcoidosis is caused by 1-α-hydroxylase production of macrophages (7) and may lead to nephrocalcinosis. One could argue that patients with nephrocalcinosis and not GIN might have a better prognosis, because under treatment, hypercalcemia is reversible and nephrocalcinosis may resolve, whereas the GIN may turn into fibrotic scars and therefore leave a permanent kidney damage.

Because of the small sample size and partially incomplete clinical data, the *p*-values for other symptoms were too large to translate them into statistically significant predictions; only the negative predictors GIN and concentration impairment were close to significance. Although all published cases of renal pediatric sarcoidosis were included, the major limitation of this analysis is the sample size, the estimation of the GFR as described in Methods and the lack of consistent definitions for the clinical symptoms in the cases. The sample size is responsible for the insignificant *p*-values and the tradeoff to choose univariate linear regression. From a statistical point of view, a multiple linear regression would have been the better choice, since it would take the influence between the initial clinical symptoms into account. Prospective registries for pediatric renal sarcoidosis with standardized data entry are needed and could verify the trends we found in this report.

Currently, no treatment protocol for pediatric renal sarcoidosis exists. In all reported cases, prednisolone was a major pillar of the treatment. Other immunosuppressants including mycophenolate mofetil, cyclophosphamide, and methotrexate were added in refractory cases or to spare steroids. Since treatment was only intensified in the severe cases, an analysis of the effect of different treatment regimens on end-of-follow-up GFR would be biased and was therefore not performed. While in the older cases only prednisolone and methylprednisolone were used, in newer cases, MTX and MMF became more important. To date, systematic evaluation of these substances in renal sarcoidosis is lacking in children as well as adults. However, in 2014, Hilderson et al. proposed a treatment protocol for renal sarcoidosis in adults (16). Similarly to what we observed in pediatric cases, prednisolone is used in induction therapy and methylprednisolone pulses are added in case of impaired renal function. If glucocorticoids fail to control the disease or cannot be tapered, other immunosuppressants are added for maintenance (14, 16). Therapeutic management remains challenging. The treatment itself has side effects and therefore is desired to be administered as short as possible, but relapses may occur during the tapering or after the end of the therapy (13). Because of multiorgan involvement in our case, interdisciplinary consensus was to start induction therapy with methylprednisolone pulse, high-dose prednisolone, and weekly oral methotrexate. Treatment was effective and remission was achieved 1 month after initiation. This is highly beneficial because studies in adults show that no significant changes in kidney function are to be expected 4–6 weeks after initiation, and therefore, early treatment success is considered a prognostic factor for a better outcome (13, 15).

## Conclusion

Renal involvement in pediatric sarcoidosis is rare but may be underreported due to variable clinical presentation and lack of systematic screening. AKI is the most common feature. Biopsy should be considered in patients with renal involvement. Presence of secondary nephrocalcinosis should be evaluated, and hypercalcemia has a trend toward a rather beneficial prognosis. In contrast, GIN in histology and concentration impairment show the strongest trend toward a decline in GFR, underscoring the importance of kidney biopsy in suspected renal sarcoidosis.

## Patient's Perspective (Translated From German)

In the beginning of the 2nd week of my stay in the children's hospital, I got the first diagnosis: cancer. Me and my whole family were shocked. The time until I was told that I had sarcoidosis and not a lymphoma was very difficult for me. By then, I had already accepted that I had cancer and was mentally prepared to repeatedly come to the hospital for a long time. Therefore, I was more than jubilant when I was told that I had sarcoidosis. Nevertheless, the following 2–3 weeks were challenging, because I still had to adapt to being sick and the whole new situation. My family, my friends, and the treating physicians supported me a lot during this difficult time.

In the beginning of the treatment, I had lots of side effects like weight gain. During that time, I did not feel comfortable, was unhappy with myself and my body. In the course of the treatment that changed to the better. Due to the dose reduction, the side effects have vanished almost completely, and I can live my life like I did before the onset of my disease.

## Data Availability Statement

The original contributions presented in the study are included in the article/Supplementary Material, further inquiries can be directed to the corresponding author.

## Ethics Statement

Written informed consent was obtained from the participant/next of kin for the publication of this case report.

## Author Contributions

RK and BL-S did the outline for the review of literature and wrote the manuscript. Literature review and data analysis and interpretation was done by RK and BL-S. AJ, MG, TS, and KA contributed to the treatment of our patient and to the manuscript. KA assessed the histology and provided the image in Figure 1. All authors contributed to the article and approved the submitted version.

## Funding

BL-S was funded by the Deutsche Forschungsgemeinschaft DFG LA1257/5-1.

## Conflict of Interest

The authors declare that the research was conducted in the absence of any commercial or financial relationships that could be construed as a potential conflict of interest.

## Publisher's Note

All claims expressed in this article are solely those of the authors and do not necessarily represent those of their affiliated organizations, or those of the publisher, the editors and the reviewers. Any product that may be evaluated in this article, or claim that may be made by its manufacturer, is not guaranteed or endorsed by the publisher.

## Supplementary Material

Supplementary Table 1 Clinical data of included and excluded patients. Patients written in italic were excluded: written in blue, exclusion occurred due to missing end-of-follow-up GFR; written in red, the patient is included in two of the collections and therefore excluded once; written in green, our criteria for renal sarcoidosis were not met. NS, not specified; ND, not determined.

Supplementary Table 2 Detailed results from univariate linear regressions. Bold printed lines are represented in Figure 2D or mentioned in the results.

## Abbreviations

AKI: acute kidney injury
AZA: azathioprine
BAL: bronchoalveolar lavage
CTX: cyclophosphamide
GIN: granulomatous interstitial nephritis
GFR: glomerular filtration rate
MMF: mycophenolate mofetil
MP: methylprednisolone
MTX: methotrexate
ND: not determined
PRED: prednisolone
ngTIN: non-granulomatous tubulointerstitial nephritis
TIN: tubulointerstitial nephritis.
